# (2*Z*)-3-(4-Fluoro­anilino)-1-(5-hy­droxy-3-methyl-1-phenyl-1*H*-pyrazol-4-yl)but-2-en-1-one

**DOI:** 10.1107/S1600536812006514

**Published:** 2012-02-17

**Authors:** Abdullah M. Asiri, Hassan M. Faidallah, Seik Weng Ng, Edward R. T. Tiekink

**Affiliations:** a Chemistry Department, Faculty of Science, King Abdulaziz University, PO Box 80203, Jeddah, Saudi Arabia; b The Center of Excellence for Advanced Materials Research, King Abdulaziz University, Jeddah, PO Box 80203, Saudi Arabia; c Department of Chemistry, University of Malaya, 50603 Kuala Lumpur, Malaysia

## Abstract

The central carbonyl group in the title compound, C_20_H_18_FN_3_O_2_, forms amine–hy­droxy N—H⋯O and hy­droxy–hy­droxy O—H⋯O hydrogen bonds, leading to two *S*(6) rings. The N-bound phenyl ring is coplanar with the five-membered ring to which it is attached [dihedral angle = 6.27 (10)°], but an overall twist in the mol­ecule is evident, the dihedral angle between the terminal phenyl and benzene rings being 27.30 (10)°. Mol­ecules aggregate into a three-dimensional architecture *via* C—H⋯F, C—H⋯O and C—H⋯π inter­actions.

## Related literature

For background to the synthesis, see: Gelin *et al.* (1983 ▶); Bendaas *et al.* (1999 ▶). For the structures of the 4-chloro and 4-meth­oxy derivatives, see: Asiri, Al-Youbi, Alamry *et al.* (2011 ▶); Asiri, Al-Youbi, Faidallah *et al.* (2011 ▶).
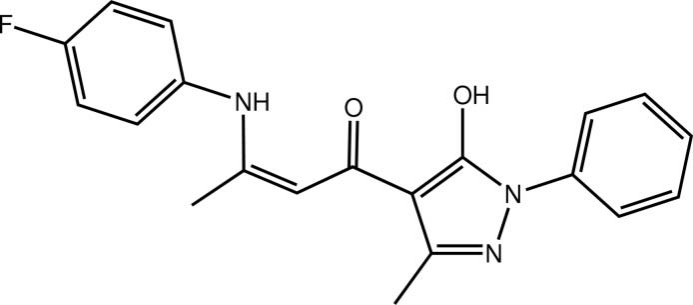


## Experimental

### 

#### Crystal data

C_20_H_18_FN_3_O_2_
*M* *_r_* = 351.37
Monoclinic, 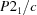

*a* = 8.3871 (7) Å
*b* = 11.1368 (9) Å
*c* = 18.4772 (16) Å
β = 101.317 (8)°
*V* = 1692.3 (2) Å^3^
*Z* = 4
Mo *K*α radiation
μ = 0.10 mm^−1^
*T* = 100 K
0.20 × 0.05 × 0.02 mm

#### Data collection

Agilent SuperNova Dual diffractometer with an Atlas detector
Absorption correction: multi-scan (*CrysAlis PRO*; Agilent, 2011 ▶) *T*_min_ = 0.981, *T*_max_ = 0.998
7099 measured reflections
3866 independent reflections
2504 reflections with *I* > 2σ(*I*)
*R*_int_ = 0.038

#### Refinement

*R*[*F* ^2^ > 2σ(*F* ^2^)] = 0.055
*wR*(*F* ^2^) = 0.123
*S* = 1.05
3866 reflections
245 parameters
2 restraints
H atoms treated by a mixture of independent and constrained refinement
Δρ_max_ = 0.25 e Å^−3^
Δρ_min_ = −0.29 e Å^−3^

### 

Data collection: *CrysAlis PRO* (Agilent, 2011 ▶); cell refinement: *CrysAlis PRO*; data reduction: *CrysAlis PRO*; program(s) used to solve structure: *SHELXS97* (Sheldrick, 2008 ▶); program(s) used to refine structure: *SHELXL97* (Sheldrick, 2008 ▶); molecular graphics: *ORTEP-3* (Farrugia, 1997 ▶) and *DIAMOND* (Brandenburg, 2006 ▶); software used to prepare material for publication: *publCIF* (Westrip, 2010 ▶).

## Supplementary Material

Crystal structure: contains datablock(s) global, I. DOI: 10.1107/S1600536812006514/hg5178sup1.cif

Structure factors: contains datablock(s) I. DOI: 10.1107/S1600536812006514/hg5178Isup2.hkl

Supplementary material file. DOI: 10.1107/S1600536812006514/hg5178Isup3.cml

Additional supplementary materials:  crystallographic information; 3D view; checkCIF report

## Figures and Tables

**Table 1:** Hydrogen-bond geometry (Å, °) *Cg*1 is the centroid of the C15–C20 benzene ring.

*D*—H⋯*A*	*D*—H	H⋯*A*	*D*⋯*A*	*D*—H⋯*A*
O1—H1⋯O2	0.86 (1)	1.73 (2)	2.521 (2)	152 (3)
N3—H3⋯O2	0.88 (1)	1.95 (2)	2.686 (2)	139 (2)
C14—H14*A*⋯F1^i^	0.98	2.43	3.309 (3)	150
C19—H19⋯O2^ii^	0.95	2.55	3.410 (3)	150
C16—H16⋯*Cg*1^iii^	0.95	2.59	3.496 (3)	161

Symmetry codes: (i) 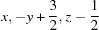; (ii) 
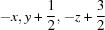
; (iii) 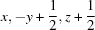.

**Fig. 1.**
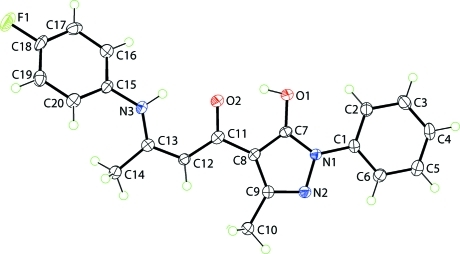
The molecular structure of (I) showing the atom-labelling scheme and displacement ellipsoids at the 50% probability level.

**Fig. 2.**
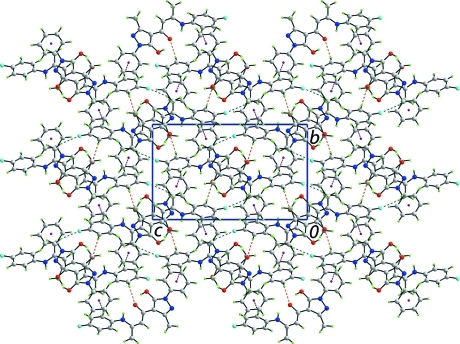
A view in projection down the a axis of the unit-cell contents of (I). The C—H···F, C—H···O and C—H···π interactions are shown as blue, orange and purple dashed lines, respectively.

## supplementary crystallographic information

### Comment

During recent investigations of reactions between pyrazoles and aniline derivatives based on literature precedents (Gelin *et al.*, 1983; Bendaas *et al.*, 1999), several compounds were isolated in crystalline form and some structures determined (Asiri, Al-Youbi, Alamry *et al.*, 2011; Asiri, Al-Youbi, Faidallah *et al.*, 2011). As a continuation of these studies, the title compound 3-(4-fluoroanilino)-1-(5-hydroxy-3-methyl-1-phenyl-1*H*-pyrazol-4-yl)but-2-en-1-one (I) was investigated.

In (I), Fig. 1, the central O2-carbonyl atom accepts two hydrogen bonds from the adjacent hydroxyl and amine groups to close a pair of fused *S*(6) rings, Table 1. The *N*-bound phenyl ring is co-planar with the five-membered ring forming a dihedral angle of 6.27 (10)°. By contrast, the fluorobenzene ring is twisted out of the plane of the rest of the molecule as seen in the value of the C13—N3—C15—C16 torsion angle of -149.5 (2)°; the dihedral angle between the terminal phenyl and benzene rings is 27.30 (10)°.

Molecules aggregate into the three-dimensional architecture *via* C—H···F, C—H···O and C—H···π interactions, Fig. 2 and Table 1.

### Experimental

A solution of 4-acetoacetyl-5-hydroxy-3-methyl-1 phenylpyrazole (0.005 mol) and 4-fluoroaniline (0.005 mol) in ethanol (25 ml) was refluxed for 2 h. The precipitate, obtained from the hot solution, was collected, washed with methanol and recrystallized from was from its ethanol-benzene solution. Orange plates were harvested; *M*.pt: 443–445 K.

### Refinement

Carbon-bound H-atoms were placed in calculated positions [C—H = 0.95 to 0.98 Å, *U*_iso_(H) = 1.2 to 1.5*U*_eq_(C)] and were included in the refinement in the riding model approximation. The N—H and O—H-atoms were located in a difference Fourier map, and were refined with distance restraints of N—H = 0.88±0.01 and O—H = 0.84±0.01 Å, respectively; their *U*_iso_ values were refined. Owing to poor agreement, the (1 7 1), (1 9 2) and (2 10 10) reflections were omitted from the final cycles of refinement.

### Crystal data

**Table:**

C_20_H_18_FN_3_O_2_	*F*(000) = 736
*M**_r_* = 351.37	*D*_x_ = 1.379 Mg m^−^^3^
Monoclinic, *P*2_1_/*c*	Mo *K*α radiation, λ = 0.71073 Å
Hall symbol: -P 2ybc	Cell parameters from 1871 reflections
*a* = 8.3871 (7) Å	θ = 2.5–27.5°
*b* = 11.1368 (9) Å	µ = 0.10 mm^−^^1^
*c* = 18.4772 (16) Å	*T* = 100 K
β = 101.317 (8)°	Plate, orange
*V* = 1692.3 (2) Å^3^	0.20 × 0.05 × 0.02 mm
*Z* = 4	

### Data collection

**Table:**

Agilent SuperNova Dual diffractometer with an Atlas detector	3866 independent reflections
Radiation source: SuperNova (Mo) X-ray Source	2504 reflections with *I* > 2σ(*I*)
Mirror monochromator	*R*_int_ = 0.038
Detector resolution: 10.4041 pixels mm^-1^	θ_max_ = 27.6°, θ_min_ = 2.5°
ω scan	*h* = −7→10
Absorption correction: multi-scan (*CrysAlis PRO*; Agilent, 2011)	*k* = −10→14
*T*_min_ = 0.981, *T*_max_ = 0.998	*l* = −22→24
7099 measured reflections	

### Refinement

**Table:**

Refinement on *F*^2^	Primary atom site location: structure-invariant direct methods
Least-squares matrix: full	Secondary atom site location: difference Fourier map
*R*[*F*^2^ > 2σ(*F*^2^)] = 0.055	Hydrogen site location: inferred from neighbouring sites
*wR*(*F*^2^) = 0.123	H atoms treated by a mixture of independent and constrained refinement
*S* = 1.05	*w* = 1/[σ^2^(*F*_o_^2^) + (0.0355*P*)^2^] where *P* = (*F*_o_^2^ + 2*F*_c_^2^)/3
3866 reflections	(Δ/σ)_max_ = 0.001
245 parameters	Δρ_max_ = 0.25 e Å^−^^3^
2 restraints	Δρ_min_ = −0.29 e Å^−^^3^

### Fractional atomic coordinates and isotropic or equivalent isotropic displacement parameters (Å^2^)

**Table:**

	*x*	*y*	*z*	*U*_iso_*/*U*_eq_	
F1	0.00255 (17)	0.62060 (12)	0.97089 (8)	0.0409 (4)	
O1	0.41629 (18)	0.25671 (13)	0.53785 (8)	0.0231 (4)	
H1	0.375 (3)	0.286 (2)	0.5730 (12)	0.069 (10)*	
O2	0.27537 (17)	0.39755 (12)	0.61177 (8)	0.0224 (4)	
N1	0.40945 (19)	0.33193 (15)	0.41680 (9)	0.0182 (4)	
N2	0.34816 (19)	0.43289 (15)	0.37538 (10)	0.0196 (4)	
N3	0.1370 (2)	0.55252 (16)	0.69228 (10)	0.0203 (4)	
H3	0.193 (2)	0.4868 (13)	0.6878 (13)	0.036 (7)*	
C1	0.4836 (2)	0.23985 (18)	0.38159 (12)	0.0180 (5)	
C2	0.5548 (2)	0.13998 (18)	0.42087 (13)	0.0241 (5)	
H2	0.5564	0.1330	0.4723	0.029*	
C3	0.6234 (3)	0.0508 (2)	0.38406 (13)	0.0277 (5)	
H3A	0.6712	−0.0177	0.4105	0.033*	
C4	0.6227 (3)	0.0607 (2)	0.30946 (13)	0.0273 (6)	
H4	0.6699	−0.0006	0.2847	0.033*	
C5	0.5528 (2)	0.1606 (2)	0.27070 (13)	0.0258 (5)	
H5	0.5520	0.1674	0.2194	0.031*	
C6	0.4842 (2)	0.25036 (19)	0.30655 (12)	0.0231 (5)	
H6	0.4376	0.3190	0.2800	0.028*	
C7	0.3767 (2)	0.33942 (18)	0.48588 (11)	0.0175 (5)	
C8	0.2937 (2)	0.44604 (18)	0.49086 (11)	0.0168 (4)	
C9	0.2795 (2)	0.49994 (18)	0.41992 (12)	0.0176 (5)	
C10	0.2000 (2)	0.61536 (18)	0.39152 (12)	0.0221 (5)	
H10A	0.1995	0.6224	0.3386	0.033*	
H10B	0.2606	0.6828	0.4178	0.033*	
H10C	0.0880	0.6166	0.3996	0.033*	
C11	0.2433 (2)	0.47650 (18)	0.55928 (12)	0.0184 (5)	
C12	0.1636 (2)	0.58608 (18)	0.56826 (11)	0.0177 (5)	
H12	0.1423	0.6388	0.5271	0.021*	
C13	0.1141 (2)	0.62287 (18)	0.63183 (12)	0.0183 (5)	
C14	0.0365 (3)	0.74430 (18)	0.63272 (12)	0.0233 (5)	
H14A	0.0672	0.7948	0.5942	0.035*	
H14B	0.0738	0.7819	0.6810	0.035*	
H14C	−0.0820	0.7354	0.6235	0.035*	
C15	0.0973 (2)	0.57223 (19)	0.76250 (12)	0.0198 (5)	
C16	0.1966 (3)	0.51939 (19)	0.82326 (13)	0.0254 (5)	
H16	0.2878	0.4734	0.8164	0.030*	
C17	0.1645 (3)	0.5328 (2)	0.89379 (13)	0.0275 (5)	
H17	0.2311	0.4954	0.9352	0.033*	
C18	0.0323 (3)	0.6024 (2)	0.90176 (13)	0.0265 (5)	
C19	−0.0693 (3)	0.6538 (2)	0.84292 (13)	0.0255 (5)	
H19	−0.1594	0.7006	0.8503	0.031*	
C20	−0.0396 (2)	0.63699 (19)	0.77237 (13)	0.0235 (5)	
H20	−0.1120	0.6694	0.7309	0.028*	

### Atomic displacement parameters (Å^2^)

**Table:**

	*U*^11^	*U*^22^	*U*^33^	*U*^12^	*U*^13^	*U*^23^
F1	0.0595 (10)	0.0460 (9)	0.0209 (8)	−0.0049 (7)	0.0172 (7)	−0.0090 (7)
O1	0.0295 (9)	0.0225 (9)	0.0180 (9)	0.0055 (7)	0.0067 (7)	0.0035 (7)
O2	0.0260 (8)	0.0237 (8)	0.0188 (9)	0.0018 (6)	0.0071 (7)	0.0027 (7)
N1	0.0188 (9)	0.0195 (9)	0.0161 (10)	0.0028 (7)	0.0031 (8)	0.0010 (8)
N2	0.0193 (9)	0.0209 (10)	0.0184 (10)	0.0018 (7)	0.0032 (8)	0.0015 (8)
N3	0.0226 (10)	0.0226 (11)	0.0166 (10)	0.0013 (8)	0.0063 (8)	−0.0013 (8)
C1	0.0131 (10)	0.0201 (12)	0.0201 (12)	0.0005 (8)	0.0013 (9)	−0.0046 (9)
C2	0.0253 (11)	0.0242 (12)	0.0224 (13)	0.0029 (10)	0.0039 (10)	−0.0031 (10)
C3	0.0292 (12)	0.0220 (13)	0.0309 (14)	0.0046 (10)	0.0035 (11)	−0.0018 (10)
C4	0.0252 (12)	0.0265 (13)	0.0300 (14)	0.0027 (10)	0.0048 (11)	−0.0121 (11)
C5	0.0204 (11)	0.0334 (14)	0.0233 (13)	0.0007 (10)	0.0036 (10)	−0.0073 (11)
C6	0.0194 (11)	0.0291 (13)	0.0202 (12)	0.0033 (9)	0.0029 (10)	−0.0004 (10)
C7	0.0159 (10)	0.0209 (11)	0.0157 (11)	−0.0022 (9)	0.0027 (9)	0.0018 (9)
C8	0.0133 (9)	0.0215 (11)	0.0157 (11)	−0.0023 (8)	0.0034 (8)	0.0001 (9)
C9	0.0150 (10)	0.0186 (11)	0.0190 (11)	−0.0020 (8)	0.0026 (9)	−0.0019 (9)
C10	0.0248 (11)	0.0213 (11)	0.0209 (12)	0.0011 (9)	0.0063 (10)	0.0005 (9)
C11	0.0139 (10)	0.0228 (12)	0.0176 (11)	−0.0063 (9)	0.0014 (9)	−0.0015 (10)
C12	0.0177 (10)	0.0195 (11)	0.0160 (11)	0.0006 (9)	0.0031 (9)	0.0008 (9)
C13	0.0164 (10)	0.0193 (11)	0.0192 (12)	−0.0044 (9)	0.0033 (9)	−0.0003 (9)
C14	0.0282 (12)	0.0227 (13)	0.0199 (13)	0.0008 (9)	0.0069 (10)	−0.0010 (10)
C15	0.0234 (11)	0.0208 (12)	0.0160 (11)	−0.0033 (9)	0.0062 (9)	−0.0013 (9)
C16	0.0283 (12)	0.0246 (13)	0.0244 (13)	0.0014 (10)	0.0082 (10)	0.0010 (10)
C17	0.0338 (13)	0.0286 (13)	0.0179 (12)	−0.0021 (11)	−0.0005 (10)	0.0035 (10)
C18	0.0391 (13)	0.0268 (13)	0.0160 (12)	−0.0091 (11)	0.0114 (11)	−0.0072 (10)
C19	0.0262 (12)	0.0243 (13)	0.0288 (14)	−0.0030 (10)	0.0126 (11)	−0.0036 (10)
C20	0.0206 (11)	0.0281 (13)	0.0213 (13)	−0.0023 (9)	0.0031 (10)	−0.0010 (10)

### Geometric parameters (Å, º)

**Table:**

F1—C18	1.364 (2)	C8—C9	1.425 (3)
O1—C7	1.325 (2)	C8—C11	1.450 (3)
O1—H1	0.860 (10)	C9—C10	1.495 (3)
O2—C11	1.298 (2)	C10—H10A	0.9800
N1—C7	1.360 (2)	C10—H10B	0.9800
N1—N2	1.400 (2)	C10—H10C	0.9800
N1—C1	1.421 (2)	C11—C12	1.417 (3)
N2—C9	1.324 (2)	C12—C13	1.382 (3)
N3—C13	1.347 (3)	C12—H12	0.9500
N3—C15	1.419 (2)	C13—C14	1.502 (3)
N3—H3	0.884 (9)	C14—H14A	0.9800
C1—C6	1.393 (3)	C14—H14B	0.9800
C1—C2	1.397 (3)	C14—H14C	0.9800
C2—C3	1.391 (3)	C15—C16	1.390 (3)
C2—H2	0.9500	C15—C20	1.397 (3)
C3—C4	1.382 (3)	C16—C17	1.389 (3)
C3—H3A	0.9500	C16—H16	0.9500
C4—C5	1.389 (3)	C17—C18	1.384 (3)
C4—H4	0.9500	C17—H17	0.9500
C5—C6	1.385 (3)	C18—C19	1.368 (3)
C5—H5	0.9500	C19—C20	1.387 (3)
C6—H6	0.9500	C19—H19	0.9500
C7—C8	1.388 (3)	C20—H20	0.9500
			
C7—O1—H1	102 (2)	H10A—C10—H10B	109.5
C7—N1—N2	110.10 (16)	C9—C10—H10C	109.5
C7—N1—C1	131.38 (18)	H10A—C10—H10C	109.5
N2—N1—C1	118.42 (16)	H10B—C10—H10C	109.5
C9—N2—N1	105.73 (17)	O2—C11—C12	121.97 (18)
C13—N3—C15	130.36 (18)	O2—C11—C8	116.11 (18)
C13—N3—H3	113.3 (15)	C12—C11—C8	121.92 (19)
C15—N3—H3	116.1 (16)	C13—C12—C11	125.6 (2)
C6—C1—C2	119.97 (19)	C13—C12—H12	117.2
C6—C1—N1	118.86 (19)	C11—C12—H12	117.2
C2—C1—N1	121.17 (19)	N3—C13—C12	120.95 (19)
C3—C2—C1	119.4 (2)	N3—C13—C14	120.42 (18)
C3—C2—H2	120.3	C12—C13—C14	118.62 (19)
C1—C2—H2	120.3	C13—C14—H14A	109.5
C4—C3—C2	120.6 (2)	C13—C14—H14B	109.5
C4—C3—H3A	119.7	H14A—C14—H14B	109.5
C2—C3—H3A	119.7	C13—C14—H14C	109.5
C3—C4—C5	119.8 (2)	H14A—C14—H14C	109.5
C3—C4—H4	120.1	H14B—C14—H14C	109.5
C5—C4—H4	120.1	C16—C15—C20	119.36 (19)
C6—C5—C4	120.3 (2)	C16—C15—N3	117.51 (18)
C6—C5—H5	119.9	C20—C15—N3	123.1 (2)
C4—C5—H5	119.9	C17—C16—C15	121.1 (2)
C5—C6—C1	119.9 (2)	C17—C16—H16	119.5
C5—C6—H6	120.0	C15—C16—H16	119.5
C1—C6—H6	120.0	C18—C17—C16	117.8 (2)
O1—C7—N1	124.71 (18)	C18—C17—H17	121.1
O1—C7—C8	127.17 (18)	C16—C17—H17	121.1
N1—C7—C8	108.11 (18)	F1—C18—C19	118.8 (2)
C7—C8—C9	104.59 (17)	F1—C18—C17	118.7 (2)
C7—C8—C11	119.57 (19)	C19—C18—C17	122.5 (2)
C9—C8—C11	135.84 (19)	C18—C19—C20	119.4 (2)
N2—C9—C8	111.47 (18)	C18—C19—H19	120.3
N2—C9—C10	119.03 (19)	C20—C19—H19	120.3
C8—C9—C10	129.50 (18)	C19—C20—C15	119.7 (2)
C9—C10—H10A	109.5	C19—C20—H20	120.1
C9—C10—H10B	109.5	C15—C20—H20	120.1
			
C7—N1—N2—C9	0.1 (2)	C11—C8—C9—N2	−179.7 (2)
C1—N1—N2—C9	176.80 (16)	C7—C8—C9—C10	179.2 (2)
C7—N1—C1—C6	172.3 (2)	C11—C8—C9—C10	−0.3 (4)
N2—N1—C1—C6	−3.6 (3)	C7—C8—C11—O2	−2.4 (3)
C7—N1—C1—C2	−7.6 (3)	C9—C8—C11—O2	177.0 (2)
N2—N1—C1—C2	176.52 (18)	C7—C8—C11—C12	177.68 (19)
C6—C1—C2—C3	−1.1 (3)	C9—C8—C11—C12	−2.9 (4)
N1—C1—C2—C3	178.82 (18)	O2—C11—C12—C13	1.2 (3)
C1—C2—C3—C4	0.5 (3)	C8—C11—C12—C13	−178.86 (19)
C2—C3—C4—C5	−0.1 (3)	C15—N3—C13—C12	179.61 (19)
C3—C4—C5—C6	0.2 (3)	C15—N3—C13—C14	0.7 (3)
C4—C5—C6—C1	−0.7 (3)	C11—C12—C13—N3	−1.2 (3)
C2—C1—C6—C5	1.2 (3)	C11—C12—C13—C14	177.71 (19)
N1—C1—C6—C5	−178.71 (17)	C13—N3—C15—C16	−149.5 (2)
N2—N1—C7—O1	178.72 (18)	C13—N3—C15—C20	33.5 (3)
C1—N1—C7—O1	2.6 (3)	C20—C15—C16—C17	−1.8 (3)
N2—N1—C7—C8	−0.3 (2)	N3—C15—C16—C17	−179.00 (19)
C1—N1—C7—C8	−176.41 (19)	C15—C16—C17—C18	−1.3 (3)
O1—C7—C8—C9	−178.6 (2)	C16—C17—C18—F1	−177.45 (19)
N1—C7—C8—C9	0.3 (2)	C16—C17—C18—C19	2.5 (3)
O1—C7—C8—C11	0.9 (3)	F1—C18—C19—C20	179.42 (18)
N1—C7—C8—C11	179.86 (17)	C17—C18—C19—C20	−0.5 (3)
N1—N2—C9—C8	0.1 (2)	C18—C19—C20—C15	−2.7 (3)
N1—N2—C9—C10	−179.39 (17)	C16—C15—C20—C19	3.8 (3)
C7—C8—C9—N2	−0.3 (2)	N3—C15—C20—C19	−179.17 (19)

### Hydrogen-bond geometry (Å, º)

Cg1 is the centroid of the C15–C20 benzene ring.

**Table:**

*D*—H···*A*	*D*—H	H···*A*	*D*···*A*	*D*—H···*A*
O1—H1···O2	0.86 (1)	1.73 (2)	2.521 (2)	152 (3)
N3—H3···O2	0.88 (1)	1.95 (2)	2.686 (2)	139 (2)
C14—H14*A*···F1^i^	0.98	2.43	3.309 (3)	150
C19—H19···O2^ii^	0.95	2.55	3.410 (3)	150
C16—H16···*Cg*1^iii^	0.95	2.59	3.496 (3)	161

Symmetry codes: (i) *x*, −*y*+3/2, *z*−1/2; (ii) −*x*, *y*+1/2, −*z*+3/2; (iii) *x*, −*y*+1/2, *z*+1/2.
